# Physician-patient communication of costs and financial burden of cancer and its treatment: a systematic review of clinical guidelines

**DOI:** 10.1186/s12885-021-08697-5

**Published:** 2021-09-16

**Authors:** Anupriya Agarwal, Ann Livingstone, Deme J. Karikios, Martin R. Stockler, Philip J. Beale, Rachael L. Morton

**Affiliations:** 1 NHMRC Clinical Trials Centre, The University of Sydney, Camperdown, NSW 2050 Australia; 2 Sydney Medical School, University of Sydney, Camperdown, New South Wales Australia; 3 Nepean Cancer Centre, Nepean Hospital, Kingswood, New South Wales Australia; 4 Concord Cancer Centre, Concord Repatriation General Hospital, Concord, New South Wales Australia; 5 Chris O’Brien Lifehouse, Camperdown, New South Wales Australia

**Keywords:** Systematic review, Guidelines, Cost discussions, Costs of care, cancer costs, Financial toxicity, Financial burden

## Abstract

**Background:**

Optimising the care of individuals with cancer without imposing significant financial burden related to their anticancer treatment is becoming increasingly difficult. The American Society of Clinical Oncology (ASCO) has recommended clinicians discuss costs of cancer care with patients to enhance shared decision-making. We sought information to guide oncologists’ discussions with patients about these costs.

**Methods:**

We searched Medline, EMBASE and clinical practice guideline databases from January 2009 to 1 June 2019 for recommendations about discussing the costs of care and financial burden. Guideline quality was assessed with the AGREE-II instrument.

**Results:**

Twenty-seven guidelines met our eligibility criteria, including 16 from ASCO (59%). 21 of 27 (78%) guidelines included recommendations about discussion or consideration of treatment costs when prescribing, with information about actual costs in four (15%). Recognition of the risk of financial burden or financial toxicity was described in 81% (22/27) of guidelines. However, only nine guidelines (33%) included information about managing the financial burden.

**Conclusions:**

Current clinical practice guidelines have little information to guide physician-patient discussions about costs of anticancer treatment and management of financial burden. This limits patients’ ability to control costs of treatment, and for the healthcare team to reduce the incidence and severity of financial burden. Current guidelines recommend clinician awareness of price variability and high costs of treatment. Clinicians are recommended to explore cost concerns and address financial worries, especially in high risk groups. Future guidelines should include advice on facilitating cost transparency discussions, with provision of cost information and resources.

**Supplementary Information:**

The online version contains supplementary material available at 10.1186/s12885-021-08697-5.

## Background

In the United States, the estimated national expenditure on cancer care in 2017 was $147.3 billion [1], and this is projected to increase with an ageing population and rise in cancer prevalence. Additionally, the advent of personalised medicine and availability of newer therapies come at increasing costs, especially as many are prescribed lifelong. As healthcare costs for cancer are higher than for other conditions [2], discussions regarding expenses are relevant and necessary to allow timely interventions that reduce the risk of financial burden. ‘Financial toxicity’, or treatment-related financial harm of cancer care has been reported in up to one in four patients with cancer [3]. This can have unintended consequences due to patients’ attempts to reduce costs by non-adherence to medication and missing healthcare appointments [4, 5]. Patients who reported higher self-rated financial burden had poorer cancer outcomes, lower quality-of-life scores, and less satisfaction with care [6].

Recognition of cancer-related financial burden has led to initiatives towards improving price transparency and value-based care in the clinical setting. In recent years, medical organisations from many countries, have recommended greater transparency about the costs of treatment. The American College of Physicians and the Australian Medical Association, both recommend that financial consent be a part of clinical care of patients [7–9]. The American Society of Clinical Oncology’s Guidance Statement on the Cost of Cancer Care [10] recommends ‘patient-physician discussions regarding the cost of care are an important component of high-quality care’. Health care providers, especially oncologists, have a greater responsibility to include discussion of costs in their communication with patients.

Enhanced patient-physician communication may heighten physicians’ awareness of financial issues and thereby help ensure patients are prescribed the most cost-effective medicines [7]. Physicians can also play a key role in educating patients to make appropriate and affordable decisions regarding their out-of-pocket costs.

A review of patient-physician costs communication indicated that the majority of patients wanted to discuss costs with their oncologists [11–13] and most physicians felt it was their responsibility. However, financial issues were not frequently addressed, and more than 70% of oncologists felt uncomfortable with such communication [14], largely due to lack of appropriate information to facilitate the discussion. Additionally, patients commonly deferred cost discussions with their oncologist until they were already experiencing financial burden [4]. However, when patients did talk about costs with their clinician, it led to lower out-of-pocket costs [15]. Clinical practice guidelines can assist physician education to direct discussions of costs and management of financial burden.

Therefore, we conducted a systematic review of published clinical practice guidelines to identify information available to oncologists regarding the discussion of costs; and the detection and management of financial burden. The motivating goal for this review was to provide a summary of existing guidelines about the discussion of costs and management of financial burden in cancer care. In addition, the review would identify gaps in the information available to guide future research and guideline development.

## Methods

The Preferred Reporting Items for Systematic Reviews and Meta-Analyses (PRISMA) [16] checklist was used to guide this review.

### Data sources and guideline selection

We conducted a systematic literature search of the following databases: PubMed/Medline, Embase, the National Guideline Clearinghouse within the Agency for Healthcare Research and Quality (AHRQ), Turning Research Into Practice (TRIP) database, Scottish Intercollegiate Guidelines Network (SIGN) and International Guidelines Network (GIN). We searched guidelines from the following organisations: American Society of Clinical Oncology (ASCO), National Comprehensive Cancer Network (NCCN), Canadian Medical Association (CMA), National Institute for Health and Care Excellence (NICE) and Cancer Australia. We limited our search to guidelines published after January 2009 following publication of the ASCO Guidance Statement for Costs of Cancer Care [10], which first proposed discussion of costs in the clinical setting. All guideline searches were finalised on 1st May 2021. Full details of the search strategy are in Table S1 in the Additional file 1.

### Study selection

Results from the five databases were downloaded into Covidence [17], a Cochrane reference management tool for systematic reviews. Two reviewers, AA and DK, independently screened the titles and abstracts. Studies considered eligible underwent full text review by AA and DK, and disagreements regarding inclusion were resolved by consensus with a third reviewer (RLM).

We selected guidelines that provided recommendations about the discussion of costs incurred by patients, the detection of financial burden, or the management of financial burden, in people with cancer. The study inclusion criteria were: 1) clinical practice guidelines endorsed by a national government or professional association, including position statements by professional associations; 2) articles about the management of advanced cancer; 3) guidelines intended for health professionals; 4) guidelines published from June 2009. We excluded guidelines that were related to the management of keratinocyte cancers, and guidelines that only included the discussion of cost-effectiveness analyses that do not apply directly to patients. No language restriction was applied.

### Guideline quality assessment

Two reviewers (AA and AL) independently applied the Appraisal of Guidelines Research and Evaluation (AGREE-II) instrument [18] to determine the methodological quality of each included guideline. The AGREE-II instrument is comprised of 23 items organised in six domains: 1. scope and purpose; 2. stakeholder involvement; 3. rigour of development; 4. clarity of presentation; 5. applicability; and 6. editorial independence. Each of the 23 items is rated on a 7-point Likert scale. Scores were calculated as a percentage (range 0 to 100%), and discrepancies between reviewers ≥4 points for an individual item were resolved by consensus. Guidelines that scored at least 60% in all domains were considered to be of adequate quality, a criterion that has been utilised in previously published guideline appraisals [19–21].

### Data extraction

We extracted the following information from clinical practice guidelines:

1. Consideration and/or discussion of direct healthcare costs incurred by patients, including provision of cost estimates when making decisions regarding treatment.
2. Recommendations for screening for financial burden or stress in patients undergoing management of cancer.
3. Recommendations for management of financial burden or financial stress in patients, including provision of support services or further information available to patients.

We summarised and collated the key recommendations in the guidelines.

## Results

### Literature search results

**Fig. 1:**
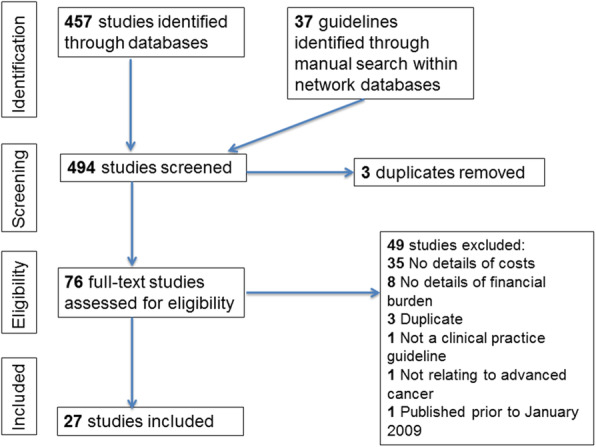
PRISMA flowchart

The literature search yielded 494 studies that underwent screening and analysis (see Fig. 1, PRISMA flowchart). After full text analysis we identified 27 guidelines that satisfied the eligibility criteria (Table 1). All included guidelines were published in English. The guidelines included in the full text analysis were from the following four groups: ASCO, SIGN, Canadian Association of Psycho-Oncology (CAPO), and NCCN.

**Table 1:** Guidelines included in the review

Guideline	Guideline organisation/ society	First author, publication year
United States		
American Society of Clinical Oncology Guidance Statement: The Cost of Cancer Care	ASCO	Meropol et al., 2009 [10]
American Society of Clinical Oncology Clinical Practice Guideline Update on Chemotherapy for Stage IV Non-Small Cell Lung Cancer	ASCO	Azzoli et al., 2009 [22]
American Society of Clinical Oncology/American Society of Hematology Clinical Practice Guideline Update on the Use of Epoetin and Darbepoetin in Adult Patients With Cancer	ASCO/ASH	Rizzo et al., 2010 [23]
Appropriate Chemotherapy Dosing for Obese Adult Patients With Cancer: American Society of Clinical Oncology Clinical Practice Guideline	ASCO	Griggs et al., 2012 [24]
Screening, Assessment, and Care of Anxiety and Depressive Symptoms in Adults with Cancer: An American Society of Clinical Oncology Guideline Adaptation	ASCO	Andersen et al., 2014 [25]
Endocrine Therapy for Hormone Receptor-Positive Metastatic Breast Cancer: American Society of Clinical Oncology Guideline	ASCO	Rugo et al., 2016 [1]
Patient-Clinician Communication: American Society of Clinical Oncology Consensus Guideline	ASCO	Gilligan et al., 2017 [26]
Anti-emetics: American Society of Clinical Oncology Clinical Practice Guideline Update	ASCO	Hesketh et al., 2017 [27]
Role of Bone-Modifying Agents in Metastatic Breast Cancer: An American Society of Clinical Oncology—Cancer Care Ontario Focused Guideline Update	ASCO/CCO	Van Poznak et al., 2017 [28]
Outpatient Management of Fever and Neutropenia in Adults Treated for Malignancy: American Society of Clinical Oncology and Infectious Diseases Society of America Clinical Practice Guideline Update	ASCO/IDSA	Taplitz et al., 2018 [29]
Fertility Preservation in Patients with Cancer: American Society of Clinical Oncology Clinical Practice Guideline Update	ASCO	Oktay et al., 2018 [30]
Metastatic Pancreatic Cancer: American Society of Clinical Oncology Clinical Practice Guideline Update	ASCO	Sohal et al., 2018 [31]
Optimizing Anticancer Therapy in Metastatic Non-Castrate Prostate Cancer: American Society of Clinical Oncology Clinical Practice Guideline	ASCO	Morris et al., 2018 [38]
Sentinel Lymph Node Biopsy and Management of Regional Lymph Nodes in Melanoma: American Society of Clinical Oncology and Society of Surgical Oncology Clinical Practice Guideline Update	ASCO/ SSO	Wong et al., 2018 [32]
Practical Assessment and Management of Vulnerabilities in Older Patients Receiving Chemotherapy: American Society of Clinical Oncology Guideline for Geriatric Oncology	ASCO	Mohile et al., 2018 [33]
Use of Larynx-Preservation Strategies in the Treatment of Laryngeal Cancer: American Society of Clinical Oncology Clinical Practice Guideline Update	ASCO	Forastiere et al., 2018 [34]
Europe		
Diagnosis and management of colorectal cancer	SIGN	Steele et al., 2011 [44] (revised 2016)
Management of lung cancer	SIGN	Fergusson et al., 2014 [46]
Management of adult testicular germ cell tumours	SIGN	Howard et al., 2011 [47]
Management of epithelial ovarian cancer	SIGN	Siddiqui et al., 2013 [45]
Cutaneous melanoma	SIGN	Brown et al., 2017 [39]
Canada		
A Pan-Canadian Clinical Practice Guideline: Assessment of Psychosocial Health Care Needs of the Adult Cancer Patient	CAPO	Howell et al., 2009 [35]
A Pan-Canadian Practice Guideline: Prevention, Screening, Assessment and Treatment of Sleep Disturbances in Adults with Cancer	CAPO	Howell et al., 2012 [36]
Pan-Canadian Practice Guideline: Screening, Assessment and Management of Psychosocial Distress, Depression and Anxiety in Adults with Cancer	CAPO	Howell et al., 2015 [37]

### Themes from guidelines

**Fig. 2:**
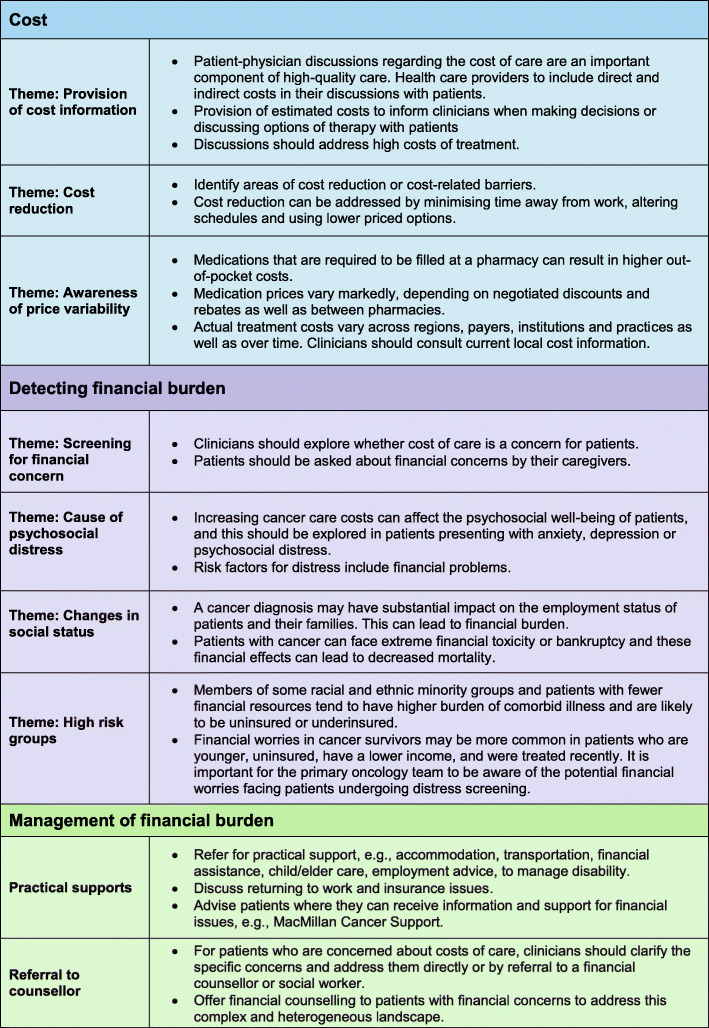
Key themes from guidelines

We identified three themes (Fig. 2) covered by the guidelines: 1. discussion of costs; 2. detection of financial burden; and 3. management of financial burden.

#### Costs

Guidelines on discussion of costs were limited to statements recommending consideration and patient awareness of high costs of care when prescribing treatments. 21 of 27 [10, 22–24, 26–42] guidelines (78%) used the term ‘costs’ within their recommendations for patient management. Of these, 16 of 21 guidelines (76%) stated clinicians should consider costs and cost concerns of patients when prescribing therapy or discussing management options. Four of 21 guidelines (19%) [22, 23, 27, 28], all from ASCO, outlined Medicare Part B/D costs to guide clinicians in when making decisions about the choice of therapy. One guideline (NCCN Palliative Care) [42] described how ‘earlier palliative care consultations have been associated with reduced healthcare costs for patients with advanced cancer and multiple comorbidities’; and a separate guideline (NCCN Older Adult Oncology) [41] recommended the use of a financial expert to discuss costs and insurance coverage options with the patient.

None of the guidelines contained recommendations or references about communication tools or the use of informed financial consent in the clinic visit. There were no summary statements provided in the guidelines to assist clinicians explain costs. However, there were sample talking points outlined in the Data Supplement to the ASCO Guideline on Systemic Therapy for Patients With Advanced Human Epidermal Growth Factor Receptor 2-Positive Breast Cancer [43] to assist clinicians explaining the costs of therapy, and links to further information about supportive services and costs.

#### Financial burden

##### Recognition of financial burden

Financial burden was recognised in 22 of 27 guidelines (81%) [10, 24–32, 35–40, 44–47]. All guidelines from SIGN (*n* = 5) contained recommendations to assess financial burden in people with cancer. Financial burden was noted in a single NCCN guideline, Distress Management [40], which recommended that clinicians consider ‘financial toxicity’ and ‘financial worries’ as a risk factor for distress. However, none of the NCCN guidelines had referral details for further support services, or management options for patients with financial burden.

Of the guidelines from ASCO, only 11 of 16 (69%) recognised financial burden as a cause of distress. The ASCO guideline on ‘Patient-Clinician Communication’ recommended clinicians ‘explore whether there are any financial constraints’ as a core communication skill that must be applied at every visit across the cancer continuum. In four of the ASCO guidelines, the discussion of financial burden was presented in the form of a generic statement: ‘When discussing financial issues and concerns, patients should be made aware of any financial counselling services that are available to address this complex and heterogeneous landscape’. However, none of these guidelines detailed any further financial counselling resources or provided further directions of where to find this information.

##### Management of financial burden

Management of financial burden was outlined in nine of 27 (33%) [25–27, 30, 39, 44–47] guidelines. This was in the form of referral to support services or financial counsellors/experts. Details of support services, for example the MacMillan Cancer Support services [48], were provided in three of the five guidelines from SIGN, with clinicians directed to refer patients to these services. Only three of the ASCO guidelines (19%; 3 of16) gave instructions to clinicians regarding management of financial concerns, and this was in the form of a ‘recommendation to refer to a financial counsellor’. One ASCO guideline, ‘Screening, Assessment and Care of Anxiety and Depressive Symptoms in Adults with Cancer’ [25], instructed clinicians to provide patients and families with education, verbal and written information regarding the availability of financial support for accommodation, drug costs, and transportation.

### Quality assessment

The AGREE-II domain scores are shown in Table S2 in the Additional file 1. The mean scores (range) for each of the domains were: scope and purpose 92% (83–100%); stakeholder involvement 83% (67–100%); rigour of development 84% (67–98%); clarity of presentation 94% (81–100%); applicability 75% (58–96%); and editorial independence 84% (79–100%).

Twenty-four (89%) guidelines were assessed as ‘recommended for use’, with appraisal scores of 5 to 7 reflecting guidelines of good to high quality. The other three (11%) guidelines were assessed as ‘recommended for use with modifications’ because scores for their appraisal were lower, primarily due to lack of reporting about their methods or limited representation of important groups on their development panels, e.g. nurses.

## Discussion

Optimising the care of patients in a financially responsible way is increasingly important in the current era of cancer care. Cancer treatment costs can influence treatment decisions by patients, and subsequently, can affect patient outcomes [49].

The issue of addressing price transparency is global and important in all healthcare systems, including those in countries with universal health care coverage. In the United States, the ‘Executive Order on Improving Price and Quality Transparency in American Healthcare to Put Patients First’ [50] has been proposed to increase price transparency and inform patients about costs of care before they make informed health care decisions. Out-of-pocket costs and rates of financial burden remain high in countries with universal health care, such as Australia, Canada, United Kingdom and countries in the European Union [51, 52]. In Australia, the Department of Health released a report supporting informed financial consent (IFC) [53], and education for consumers and specialists regarding the costs of healthcare. The Australian Medical Association and specialist colleges have also supported IFC and the provision to patients of information about medical fees [7].

Research also shows that interventions to address ‘pricing failure’, i.e., non-disclosure of prices to patients, can reduce waste in healthcare. It has been estimated that greater transparency about pricing for office and laboratory visits in the US could result in healthcare savings of $29 billion USD [54].

Limited information is contained in the guidelines for oncologists; however, there are some key recommendations that appear consistently: 1. Clinician awareness of price variability between treatment options, insurance coverage and geographical regions; 2. Screening for financial stress as a cause of psychological distress, especially in high risk groups such as low socioeconomic status populations and ethnic minority groups; 3. Referral to practical supports and services to alleviate financial burden.

The findings of our systematic review reveal a paucity of information for clinicians about communication regarding the costs of treatment, recognition of financial burden, and the management of patients’ financial burden. Most guidelines recommended costs be considered and discussed. However, there was little information to guide these discussions in the clinical setting. In addition, although there were recommendations to refer patients to support services, there was limited information about how or where to find these support services.

Clinicians have described multiple barriers to discussing costs, primarily time constraints and perceived absence of viable solutions if costs are a concern [55, 56]. Provision of information regarding costs and support services within guidelines may reduce these barriers and facilitate the discussion in the clinical setting. In addition, incorporation of multi-disciplinary team members such as nurses, social workers, counsellors, and pharmacists as referral services for patients to discuss costs and financial concerns may assist management. An outline of questions that providers can consider asking their patients during the initial and follow-up consultations is provided in Table 2.

**Table 2:** A guide for clinicians to improve cost communication

Time	Costs of care	Financial burden
At initial appointment	● Discuss options of therapy, with estimates of costs ● Provide information on fees to attend services, e.g., healthcare appointments, hospital services	● Discuss patient employment, insurance coverage and household income status ● Suggest local and national support services if available ● Consider early introduction to financial navigator to develop a financial plan
At follow-up appointments	● Discuss costs of treatment when prescribing new therapies ● Discuss ability to afford current prescribed therapy ● Assess for presence of cost-saving strategies, e.g., skipping doses or appointments	● Screen for presence of psychosocial issues arising from financial burden ● Explore ability to work and patient’s support network ● Consider referral to financial navigator or counsellor

This is the first systematic review about information regarding costs of cancer care in published clinical practice guidelines. The literature review included international guidelines with no language restrictions to ensure the generalisability of our findings. We undertook a comprehensive search of guideline databases, including manual searches. These results are particularly valuable for health care professionals caring for patients with cancer, especially oncologists and cancer nurses. They will be of special consideration to cancer societies and organisations that develop and publish these guidelines.

One limitation of our review is that we restricted it to guidelines about the management of advanced cancers. This is not meant to downplay the considerable financial burden faced by survivors of cancer treated with curative intent, which may also have long-term effects on wellbeing. High medical costs have been reported to cause psychological distress in 34% of cancer survivors, and disproportionately affect those without insurance [57]. Our decision to include only guidelines relating to the management of advanced cancer was to keep the recommendations regarding costs and financial burden consistent with changes in employment and work status. These changes are more likely to be permanent in people with advanced cancers.

Our review demonstrates that there is limited information to guide clinicians on how to discuss the costs and financial impact of cancer treatment with their patients. The study identifies gaps in recommendations and resources that should be used to guide future research and development of guidelines to make this information accessible to clinicians (See Table 3). Future guidelines should contain more information about the optimal timing, frequency, and content of these discussions. In addition, future guidelines should include more advice on how oncologists should explain the costs of care accurately and transparently, along with suggestions to reduce financial burden.

**Table 3:** Current gaps in literature to guide clinicians on communication of costs and financial burden

Theme	Gaps	Unanswered questions
Discussion of costs	Timing of discussion	When is the best time to bring up costs of care?
	Person responsible	Who is the best/most appropriate healthcare provider to discuss costs?
	Resources for costs information	What resources should healthcare providers use to access costs information to inform patients?
Screening for financial burden	Optimal time to screen	When is the best time to screen patients for presence of financial burden?
	Ideal tool e.g. survey, questionnaire to screen for financial burden	What is the best tool to use for screening of financial issues?
Managing financial burden	Resources and support materials	What are the resources and support available to patients and families to relieve financial burden?
	Person responsible to act upon financial burden	Who is the best/most appropriate healthcare provider to discuss management and provide financial counselling?
	Region-specific or country-specific guidelines	What financial assistance or practical supports are available for patients to use in a particular region/area/health service?

Our review demonstrates a scarcity of information and guidelines from other major cancer networks and countries where the rising costs of cancer therapy are becoming an increasing problem. This review highlights findings from primary studies indicating that starting conversations early about the costs of cancer and its treatment may provide patients with a greater sense of control over their management, and reduce both the incidence and severity of financial burden.

Clinical practice guidelines for discussion about cancer costs could be improved by: first, providing costs estimates to clinicians to enable the discussion of treatment options with patients; second, providing information to clinicians about how to identify individuals at high risk of developing financial burden; and third, discussing strategies and options for management of financial burden, including the provision of details of support services. Further research is needed to assess the acceptability and feasibility of clinician-initiated cost discussions in the clinic setting. Further studies on the facilitators and barriers of communication about costs will allow the development of improved guidelines and increase clinician uptake of the recommendations.

## Conclusion

Current clinical practice guidelines have limited information to guide discussions about the costs of anticancer treatment and the management of financial burden. This limits the ability of patients to control the costs of their cancer management, and of the healthcare team to reduce the incidence and severity of financial burden.

## Supplementary Information

**Additional file 1: Table S1.** Search terms used in Ovid Online. **Table S2.** Domain Scores (%) of the included clinical practice guidelines using the AGREE II instrument.

## Data Availability

The datasets used and/or analysed during the current study are available from the corresponding author on reasonable request.

## Abbreviations

ASCO: American Society of Clinical Oncology
NCCN: National Comprehensive Cancer Network
SIGN: Scottish Intercollegiate Guidelines Network
GIN: Guidelines International Network
CMA: Canadian Medical Association
NICE: National Institute for Health and Care Excellence
