# Supplementary material for: Physician-patient communication of costs and financial burden of cancer and its treatment: a systematic review of clinical guidelines
Source: BMC Cancer. 2021 Sep 16;21:1036. doi: 10.1186/s12885-021-08697-5 (PMC8447743; doi:10.1186/s12885-021-08697-5)
Supplement: Supplementary file 1 — Additional file 1: Table S1. Search terms used in Ovid Online. Table S2. Domain Scores (%) of the included clinical practice guidelines using the AGREE II instrument. [file 12885_2021_8697_MOESM1_ESM.docx]

Table S1: Search terms used in Ovid Online:

| Ovid MEDLINE(R) <1946 to July Week 4 2019> | | | |
| --- | --- | --- | --- |
| **#** | **Search Statement** | **Results** | **Annotation** |
| 1 | practice guideline/ | 23651 |  |
| 2 | ((guideline* or statement*) adj4 (practice* or clinic*)).tw. | 39279 |  |
| 3 | 1 or 2 | 58372 |  |
| 4 | exp Neoplasm Metastasis/ | 184789 |  |
| 5 | neoplasms/ or neoplasms by histologic type/ or "neoplasms, complex and mixed"/ or "neoplasms, connective and soft tissue"/ or "neoplasms, germ cell and embryonal"/ or "neoplasms, glandular and epithelial"/ or neoplasms, gonadal tissue/ or neoplasms, nerve tissue/ or neoplasms, vascular tissue/ or "nevi and melanomas"/ or neoplasms by site/ or abdominal neoplasms/ or anal gland neoplasms/ or bone neoplasms/ or breast neoplasms/ or digestive system neoplasms/ or endocrine gland neoplasms/ or eye neoplasms/ or "head and neck neoplasms"/ or nervous system neoplasms/ or pelvic neoplasms/ or skin neoplasms/ or soft tissue neoplasms/ or splenic neoplasms/ or thoracic neoplasms/ or urogenital neoplasms/ or neoplasms, hormone-dependent/ or neoplasms, multiple primary/ or multiple endocrine neoplasia/ or neoplasms, radiation-induced/ or neoplasms, second primary/ or neoplastic processes/ or neoplasm metastasis/ or neoplasm recurrence, local/ or neoplasm, residual/ or neoplastic syndromes, hereditary/ or paraneoplastic syndromes/ | 1069865 |  |
| 6 | [metasta*.tw.](http://scanmail.trustwave.com/?c=1688&d=55-C2Vt6fmCXekE_qWalw2z083ibpgms-buI84BLyg&u=http%3a%2f%2fmetasta%2a%2etw) | 370481 |  |
| 7 | 4 or 5 or 6 | 1285523 |  |
| 8 | 3 and 7 | 4607 |  |
| 9 | financial [impact.mp.](http://scanmail.trustwave.com/?c=1688&d=55-C2Vt6fmCXekE_qWalw2z083ibpgms-bbY94NCxg&u=http%3a%2f%2fimpact%2emp) | 1364 |  |
| 10 | "cost of illness"/ | 23037 |  |
| 11 | ((patient* or person* or famil*) adj4 (cost* or financ* or burden* or stress*)).tw. | 72893 |  |
| 12 | 10 or 11 | 91755 |  |
| 13 | 8 and 12 | 78 |  |

Table S2: Domain Scores (%) of the included clinical practice guidelines using the AGREE II instrument

|  | Guideline Title | Organisation (Year) | Domain 1 (Scope and Purpose) | Domain 2 (Stakeholder Involvement) | Domain 3 (Rigour of Development) | Domain 4 (Clarity of Presentation) | Domain 5 (Applicability) | Domain 6 (Editorial Independence) | | Overall Quality Assessment Rating (Comment) |
| --- | --- | --- | --- | --- | --- | --- | --- | --- | --- | --- |
|  |  |  |  |  |  |  |  |  | |  |
|  | ASCO Guidelines | | | | | | | | | |
| 1 | Guidance statement on cost of cancer | ASCO | 97% | 67% | 67% | 81% | 79% | 92% | | 75% - Recommended |
| 2 | Patient-clinician communication | ASCO | 92% | 86% | 92% | 92% | 79% | 92% | | 92% - Recommended |
| 3 | Update for chemotherapy in stage IV NSCLC | ASCO (2009) | 86% | 50% | 71% | 97% | 60% | 83% | | 75% - Recommended |
| 4 | Antiemetics | ASCO | 100% | 97% | 91% | 97% | 83% | 83% | | 92% - Recommended |
| 5 | Appropriate chemotherapy dosing for obese adults | ASCO | 100% | 94% | 79% | 100% | 58% | 88% | | 83% |
| 6 | Update on use of epoeitin and darbapoeitin in cancer | ASCO/ASH | 92% | 78% | 73% | 89% | 65% | 75% | | 75% - Recommended with modifications.  Appraiser 1: Recommend more info re advisory group composition and alternative way to display recommendations as flow chart or table. Appraiser 2: More information on methodology would be appropriate. |
| 7 | Fertility preservation in patients with cancer | ASCO | 100% | 89% | 94% | 100% | 67% | 83% | | 83% - Recommended |
| 8 | Endocrine therapy for hormone receptor positive metastatic breast cancer | ASCO | 100% | 94% | 97% | 100% | 81% | 83% | | 92% - Recommended |
| 9 | Metastatic pancreatic cancer | ASCO | 100% | 94% | 98% | 100% | 81% | 88% | | 92% - Recommended |
| 10 | Optimising anticancer therapy in metastatic castration resistant prostate cancer | ASCO | 100% | 92% | 92% | 100% | 67% | 88% | | 83% - Recommended |
| 11 | Outpatient management of fever and neutropaenia in adults treated for malignancy | ASCO | 100% | 97% | 91% | 100% | 83% | 83% | | 92% - Recommended |
| 12 | Practical assessment and management of vulnerabilities in older patients | ASCO | 100% | 94% | 88% | 97% | 65% | 83% | | 83% - Recommended |
| 13 | Screening, assessment, and care of anxiety and depressive symptoms in adults with cancer | ASCO | 100% | 94% | 89% | 100% | 75% | 83% | | 83% - Recommended |
| 14 | Sentinel lymph node biopsy and management of regional nodes in melanoma | ASCO | 97% | 89% | 94% | 100% | 85% | 83% | | 92% - Recommended |
| 15 | Use of larynx preserving strategies in laryngeal cancer | ASCO | 100% | 89% | 91% | 94% | 73% | 79% | | 92% - Recommended |
| 16 | Bone modifying agents in metastatic breast cancer | ASCO | 94% | 75% | 88% | 92% | 71% | 79% | | 75% - Recommended with modifications.  Appraiser 1: This is an update tailored to answering 2 clinical questions, so not very rigorous methodology. But a good guideline Appraiser 2: Consider addition of other panel experts? Nurses |
|  | SIGN Guidelines | | | | | | | | | |
| 17 | Colorectal cancer | SIGN | 94% | 97% | 92% | 100% | 88% | 83% | | 83% - Recommended |
| 18 | Epithelial ovarian cancer | SIGN | 94% | 100% | 85% | 97% | 90% | 83% | | 92% - Recommended |
| 19 | Lung cancer | SIGN | 97% | 100% | 83% | 97% | 85% | 88% | | 92% - Recommended |
| 20 | Testicular cancer | SIGN | 100% | 100% | 85% | 100% | 85% | 83% | | 92% - Recommended |
| 21 | Cutaneous melanoma | SIGN |  |  |  |  |  |  | |  |
|  | CAPO Guidelines | | | | | | | | | |
| 22 | Screening, assessment and management of psychosocial distress, depression and anxiety in adults with cancer | CAPO | 92% | 72% | 91% | 94% | 79% | 100% | | 83% - Recommended |
| 23 | Sleep disturbances in adults with cancer | CAPO | 100% | 75% | 97% | 100% | 77% | 100% | | 92% - Recommended |
| 24 | Psychosocial healthcare needs | CAPO | 100% | 72% | 96% | 100% | 83% | 100% | | 92% - Recommended |
|  | NCCN Guidelines | | | | | | | | | |
| 25 | NCCN older adult oncology | NCCN | 89% | 67% | 85% | 100% | 96% | 100% | 92% - Recommended | |
| 26 | Distress management | NCCN | 83% | 94% | 73% | 100% | 92% | 100% | 83% - Recommended | |
| 27 | Palliative care | NCCN | 83% | 72% | 81% | 94% | 83% | 91% | 83% - Recommended | |
|  |  |  |  |  |  |  |  |  |  | |
|  |  |  |  |  |  |  |  |  |  | |
|  | **Average Scores** |  | **92.20%** | **82.50%** | **83.80%** | **93.40%** | **75.20%** | **84%** |  | |
